# Knockdown of Amyloid Precursor Protein Increases Ion Channel Expression and Alters Ca^2+^ Signaling Pathways

**DOI:** 10.3390/ijms24032302

**Published:** 2023-01-24

**Authors:** Maria Paschou, Danai Liaropoulou, Vasileia Kalaitzaki, Spiros Efthimiopoulos, Panagiota Papazafiri

**Affiliations:** 1 Division of Animal and Human Physiology, Department of Biology, National and Kapodistrian University of Athens, 15784 Athens, Greece; 2 Institute of Laboratory Animal Science, University of Zurich, 8952 Schlieren, Switzerland

**Keywords:** amyloid precursor protein, ampa receptors, ryanodine receptors, inositol 1,4,5-triphosphate receptors, STIM1, p-AKT, Ca^2+^ signaling

## Abstract

Although the physiological role of the full-length Amyloid Precursor Protein (APP) and its proteolytic fragments remains unclear, they are definitively crucial for normal synaptic function. Herein, we report that the downregulation of APP in SH-SY5Y cells, using short hairpin RNA (shRNA), alters the expression pattern of several ion channels and signaling proteins that are involved in synaptic and Ca^2+^ signaling. Specifically, the levels of GluR2 and GluR4 subunits of the alpha-amino-3-hydroxy-5-methyl-4-isoxazolepropionic acid glutamate receptors (AMPAR) were significantly increased with *APP* knockdown. Similarly, the expression of the majority of endoplasmic reticulum (ER) residing proteins, such as the ER Ca^2+^ channels IP_3_R (Inositol 1,4,5-triphosphate Receptor) and RyR (Ryanodine Receptor), the Ca^2+^ pump SERCA2 (Sarco/endoplasmic reticulum Ca^2+^ ATPase 2) and the ER Ca^2+^ sensor STIM1 (Stromal Interaction Molecule 1) was upregulated. A shift towards the upregulation of p-AKT, p-PP2A, and p-CaMKIV and the downregulation of p-GSK, p-ERK1/2, p-CaMKII, and p-CREB was observed, interconnecting Ca^2+^ signal transduction from the plasma membrane and ER to the nucleus. Interestingly, we detected reduced responses to several physiological stimuli, with the most prominent being the ineffectiveness of SH-SY5Y/APP- cells to mobilize Ca^2+^ from the ER upon carbachol-induced Ca^2+^ release through IP_3_Rs and RyRs. Our data further support an emerging yet perplexing role of APP within a functional molecular network of membrane and cytoplasmic proteins implicated in Ca^2+^ signaling.

## 1. Introduction

Amyloid Precursor Protein (APP) is a type I transmembrane protein mainly known as the precursor of amyloid-β (Aβ) peptide, the main component of the plaques found in patients affected by Alzheimer’s disease (AD). *APP* was first cloned 30 years ago [1,2], and since then, our understanding of the structure of the *APP* gene and protein function in synaptic formation and stability has progressed considerably. Specifically, it was found that three major APP isoforms (APP695, APP751, APP770) emanate from differential splicing of exons 7 and 8 [3], while APP695 is the main isoform found in neurons. Moreover, studies conducted on *APP* knockdown in mice and primary neurons revealed many functions of the full-length protein at the synapse [4,5], including transcriptional regulation, plasticity, and neuroprotection, through interactions with a wide range of proteins [6]. Precisely, it has been shown that APP is targeted to presynaptic terminals [7,8] and dendrites [9], where it plays a role in the formation, maintenance, and function of synapses [10,11,12]. Knockout of *APP* in GABAergic neurons leads to an elevation of Ca_v_1.2, the pore-forming subunit of L-type Voltage-gated Ca^2+^ channels (L-VGCCs), and subsequent rises in Ca^2+^ currents that can be reversed by the reintroduction of *APP* [13]. The involvement of APP in Ca^2+^ homeostasis is further supported by studies reporting that expression of human APP in rat cortical neurons increases L-VGCC currents [14] and controls store-operated Ca^2+^ entry [15]. Moreover, APP interacts with Homer2 and Homer3, which play important roles in Ca^2+^ homeostasis [16]. Notably, *APPswe* mutation leads to increased frequency of spontaneous Ca^2+^-oscillations in rat hippocampal neurons [17], while in fibroblasts isolated from *APP*−/− mice, inositol 1,4,5-triphosphate (IP_3_) -induced Ca^2+^ efflux from the endoplasmic reticulum (ER) is significantly reduced [18]. In this direction, we have previously shown that downregulation of APP in neuroblastoma SH-SY5Y cells interferes with Store-Operated Ca^2+^ channel (SOC) activity and enhances both ER and acidic store Ca^2+^ content, all resulting in the upregulation of Ca^2+^-based signaling networks [19]. 

Intracellular Ca^2+^ rise is immediately sensed by calmodulin that activates Ca^2+^/Calmodulin-dependent kinases (CaMKs), such as CaMKII and CaMKIV [20], and extracellular signal-regulated kinases 1/2 (ERK1/2), ultimately leading to the phosphorylation and subsequent activation of cyclic AMP response element binding protein (CREB) in the nucleus [21,22]. Therefore, these Ca^2+^ signaling molecules may represent regulatory targets for APP and can be mediators of APP function in the synapse. Noticeably, using a conditional *APP*-transgenic mouse model, Born et al. managed to rescue hypersynchronous network activity, which characterizes epileptogenesis in AD [23]. The latter study is of particular importance as it revealed that the Aβ and full-length APP effects could be dissociated, underscoring a role for the full-length APP itself in synaptic activity. In neurons, synaptic plasticity is also supported by Phosphatidylinositol-3 kinase/AKT (PI3K/AKT) signaling in response to several neurotrophic factors, which have a crucial role in the regulation of receptor trafficking [24]. Interestingly, this signaling pathway is attenuated by Aβ oligomers, while several synthetic or natural compounds that stimulate PI3K/AKT pathway have beneficial impacts on AD [25]. 

To elucidate the functional importance of the full-length APP in Ca^2+^-dependent signaling pathways, we previously employed cortical brain slices of wildtype C57bl/6 mice and *APP*−/− C57bl/6 mice and compared depolarization-induced activation of the kinases ERK1/2 and CaMKII [26]. Our results indicated that the absence of APP inhibited the depolarization-induced increase in the phosphorylation levels of ERK1/2 and CaMKII. Similarly, in the present study, we used human neuroblastoma wild-type (wt) SH-SY5Y cells and SH-SY5Y/APP- cells, the latter stably expressing the short hairpin RNA (shRNA) targeting the coding region of *APP* gene and set out to investigate the expression profiles of several proteins involved in synaptic signaling. In addition, we used the SK-N-SH cell line, from which SH-SY5Y cells have originated, exhibiting moderate levels of APP. We provide evidence for alterations in the levels of the alpha-amino-3-hydroxy-5-methyl-4-isoxazolepropionic acid (AMPA) receptor subunits GluR2 and GluR4, the IP_3_R2, IP_3_R3, RyR1 and RyR2 subunits of ER Ca^2+^ channels, as well as the Sarco/endoplasmic reticulum Ca^2+^ ATPase 2 (SERCA2) and the ER Ca^2+^ sensor STIM1 (Stromal Interaction Molecule 1). Furthermore, we observed increased phosphorylation of the alternative appellation for Protein kinase B (p-AKT), protein phosphatase 2A (p-PP2A), and p-CaMKIV and impaired responses to several physiological stimuli. 

## 2. Results

Herein, we examined the effects of APP downregulation on the levels of key signal transduction proteins that govern Ca^2+^ -mediated signaling from the plasma membrane to the nucleus during synaptic function. In particular, we compared their profile in SHSY5Y/APP- cells to the respective SH-SY5Y cells. To further evaluate the implication of APP, SK-N-SH cells, which moderately express APP, were also employed. 

### 2.1. Effect of APP Downregulation on Plasma Membrane Proteins

**Figure 1:**
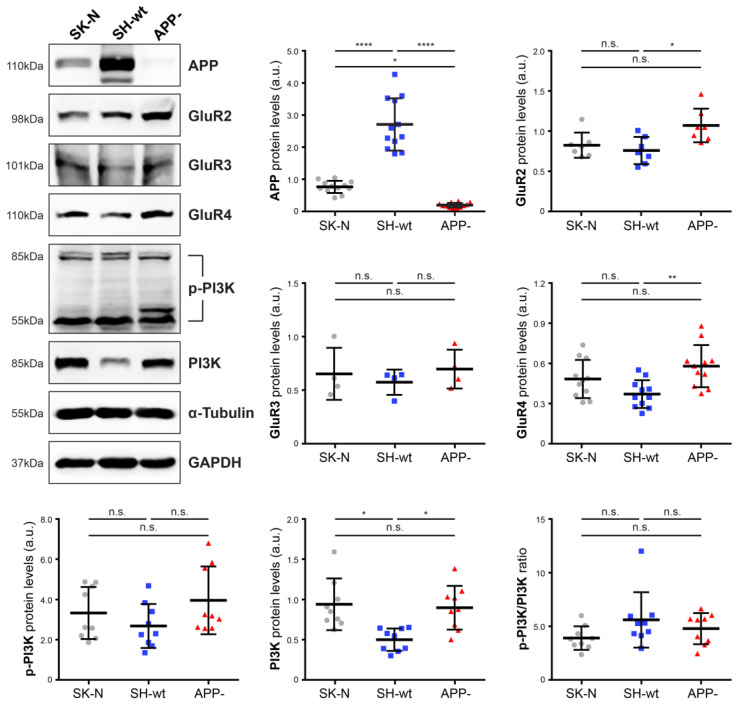
Effects of APP downregulation on plasma membrane proteins. Representative immunoblots and quantification of the levels of APP, GluR2-4, PI3K, and its phosphorylated forms in total protein extracts (50 μg/lane) from SH-SY5Y (SH-wt), SH-SY5Y/APP- (APP-) and SK-N-SH (SK-N) cells. GAPDH or α-Tubulin were used as loading controls for normalization. The molecular weights of the proteins are depicted on the left of each blot image. The levels of p-PI3K are also expressed as the ratio of the pixel intensity of the phosphorylated bands to the intensity of total PI3K. Data are presented as arbitrary units (a.u.) of the mean ± S.D. (*n* = 4–11). Dots represent individual data points. Statistical significance was evaluated by one-way ANOVA followed by Tukey’s multiple comparison tests (* *p* < 0.05, ** *p* < 0.01, **** *p* < 0.0001). APP, Amyloid Precursor Protein; GluR, Glutamate receptor subunit; Phosphatidylinositol-3 kinase (PI3K); GAPDH, Glyceraldehyde 3-phosphate dehydrogenase.

First, we investigated the effect of APP downregulation on the levels of proteins located at the plasma membrane. Total protein extracts from cell lines expressing different APP levels (Figure 1), i.e., SK-N-SH, SH-SY5Y, and SH-SY5Y/APP− cells, were analyzed using western blot for three members of the AMPA subfamily of ionotropic glutamate receptors (GluR2, GluR3, GluR4) and PI3K. Among GluRs, GluR3 levels are similar between the three cell lines, while GluR2 and GluR4 levels are significantly increased in SH-SY5Y/APP− cells, compared to SH-SY5Y [41.21 ± 26.65% (*p* < 0.05) and 62.58 ± 47.13% (*p* < 0.01) increase, respectively]. GluR1 endogenous levels were not detectable in any of the cell lines used. PI3K is a group of plasma membrane lipid kinases consisting of three subunits: the p85 and p55 regulatory subunits and the p110 catalytic subunit. Our results revealed that total levels of PI3K (85kDa) are increased in SH-SY5Y/APP− cells by 70.38 ± 54.59% (*p* < 0.05), compared to SH-SY5Y, while the levels of the phosphorylated subunits (p85 and p55) do not differ significantly between these cells. Interestingly though, an additional band (approximately at 60 kDa) was detected in SH-SY5Y/APP− cells, which was considered for quantification. Calculation of the ratio of the pixel intensity of the phosphorylated bands to the intensity of the total protein yielded no significant differences. 

### 2.2. Effects of APP Downregulation on Selected Components of Signaling Pathways

**Figure 2:**
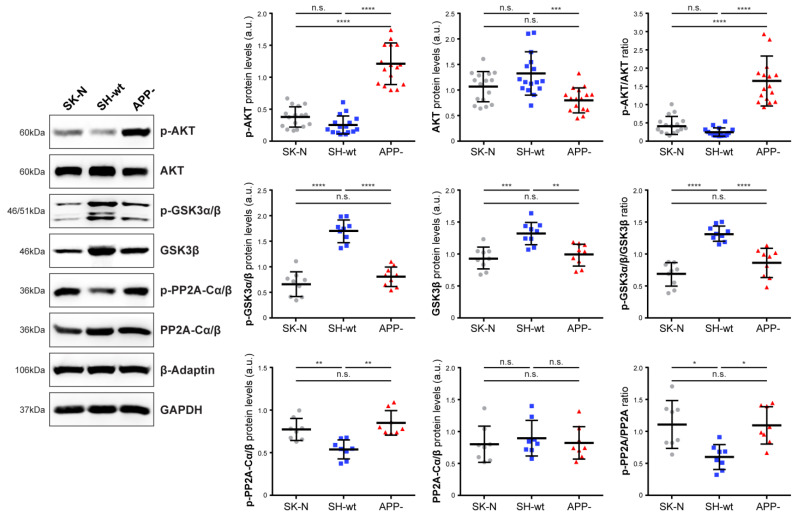
APP downregulation induces AKT and PP2A phosphorylation. Representative immunoblots and quantification of the levels of AKT, GSK3α/β, PP2A, and their phosphorylated forms in total protein extracts (50 μg/lane) from SH-SY5Y (SH-wt), SH-SY5Y/APP- (APP-), and SK-N-SH (SK-N) cells. GAPDH or β-Adaptin was used as loading controls for normalization. The molecular weights of the proteins are depicted on the left of each blot image. The levels of p-AKT, p-GSKα/β and p-PP2A-Cα/β are expressed as the ratio of the pixel intensity of the phosphorylated proteins to the intensity of total AKT, GSK3α/β and PP2A-Cα/β. Data are presented as arbitrary units (a.u.) of the mean ± S.D. (*n* = 8–17). Dots represent individual data points. Statistical significance was evaluated by one-way ANOVA followed by Tukey’s multiple comparison tests (* *p* < 0.05, ** *p* < 0.01, *** *p* < 0.001, **** *p* < 0.0001). AKT, Alternative appellation for Protein kinase B; GSK3, Glycogen synthase kinase-3; PP2A-Cα/β, Protein phosphatase 2A-C alpha/beta; GAPDH, Glyceraldehyde 3-phosphate dehydrogenase.

**Figure 3:**
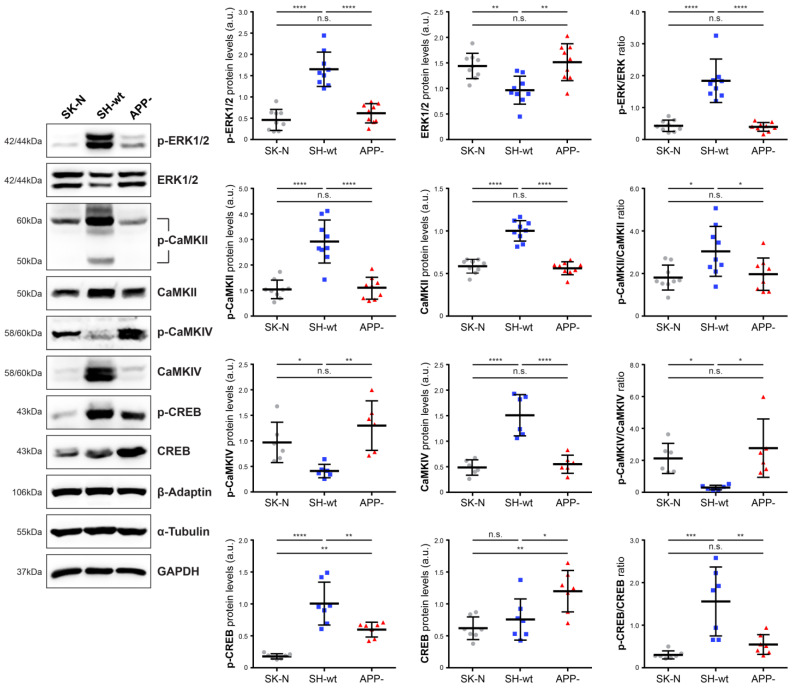
APP downregulation diminishes p-ERK1/2, p-CaMKII, and p-CREB levels and increases CaMKIV phosphorylation. Representative immunoblots and quantification of the levels of ERK1/2, CaMKII, CaMKIV, and CREB and their phosphorylated forms in total protein extracts (50 μg/lane) from SH-SY5Y (SH-wt), SH-SY5Y/APP- (APP-), and SK-N-SH (SK-N) cells. GAPDH, α-Tubulin, and β-Adaptin were used as loading controls for normalization. The molecular weights of the proteins are depicted on the left of each blot image. The levels of p-ERK1/2, p-CaMKII, p-CaMKIV, and p-CREB are expressed as the ratio of the pixel intensity of the phosphorylated bands to the intensity of total ERK1/2, CaMKII, CaMKIV, and CREB. Data are presented as arbitrary units (a.u.) of the mean ± S.D. (*n* = 7–9). Dots represent individual data points. Statistical significance was evaluated by one-way ANOVA followed by Tukey’s multiple comparison tests (* *p* < 0.05, ** *p* < 0.01, *** *p* < 0.001, **** *p* < 0.0001). ERK1/2, extracellular signal-regulated kinases 1/2; CAMKII, Ca^2+^/calmodulin dependent kinase II; CAMKIV, Ca^2+^/calmodulin dependent kinase IV; CREB, cyclic AMP response element binding protein; GAPDH, Glyceraldehyde 3-phosphate dehydrogenase.

We next examined the endogenous levels of un-phosphorylated and phosphorylated forms of various signaling molecules, mainly kinases, and the results are grouped in Figure 2 and Figure 3. Western blot analysis revealed that the levels of p-AKT are significantly increased in SH-SY5Y/APP- cells [approximately 5-fold (*p* < 0.001) increase compared to SH-SY5Y and 3.3-fold (*p* < 0.001) increase compared to SK-N-SH] (Figure 2). On the contrary, AKT levels are decreased by 39.39 ± 18.88% (*p* < 0.001) in SH-SY5Y/APP- cells leading to a 7-fold (*p* < 0.001) increase in the pAKT/AKT ratio, compared to SH-SY5Y cells.

Glycogen synthase kinase-3 (GSK3) has two isoforms, GSK3α and GSK3β, and phosphorylation of GSK3α at serine-21 or GSK3β at serine-9 inhibits the kinase activity. Several signaling pathways, mediated by AKT, protein kinase A (PKA), protein kinase C (PKC), and other kinases, induce the phosphorylation of GSK3 at both sites [27]. Remarkably, our results show that p-GSK3α/β levels in SH-SY5Y/APP- are reduced by 52.63 *±* 11.35% (*p* < 0.001) compared to SH-SY5Y, despite the increased levels of p-AKT (Figure 2). A decrease was also observed in the total levels of GSK3β (*p* < 0.01), leading to a 34.96 *±* 17.17% (*p* < 0.001) decrease in the p-GSK3/GSK3 ratio. This finding indicates that another kinase or a phosphatase may be involved in this signaling network. In this direction, active protein phosphatase 2A (PP2A) can dephosphorylate GSK3 to activate it or, inversely, activated GSK3β can induce PP2A inactivation via phosphorylation at Tyr307, a modification that can be detected by the specific antibody used in the present study. Our results indicate that the latter mechanism could be responsible for p-GSK3α/β downregulation in SH-SY5Y/APP- cells. Specifically, we observed that p-PP2A-Cα/β levels are higher in SH-SY5Y/APP- [up to 152.18 ± 28.94% (*p* < 0.01) compared to SH-SY5Y] and in SK-N-SH cells [up to 150.68 ± 25.90% (*p* < 0.01) compared to SH-SY5Y], in which p-GSK3α/β levels are relatively low. Furthermore, inactivated PP2A (elevated p-PP2A-Cα/β) could account for the sustained phosphorylation of AKT in SH-SY5Y/APP- cells. 

Significant differences were also found in the second group (Figure 3), which includes proteins considered to play a crucial role in synaptic Ca^2+^ signaling. Specifically, among the un-phosphorylated forms, the levels of ERK1/2 and CREB are significantly higher in SH-SY5Y/APP- cells [up to 156.84 ± 37.43% (*p* < 0.01) and 158,40 ± 42,64% (*p* < 0.05), respectively, compared to SH-SY5Y]. Conversely, CaMKII and CaMKIV levels are decreased by 43.80 ± 7.56% (*p* < 0.001) and 63.62 ± 11.79% (*p* < 0.001) in SH-SY5Y/APP-, respectively, compared to SH-SY5Y, with CaMKIV levels exhibiting the most prominent increase. 

However, except for CaMKII, the phosphorylation of which also decreases in SH-SY5Y/APP- cells, the levels of the phosphorylated form of ERK1/2 or CREB are inversely correlated to the respective total levels; i.e., when total levels decrease, the activated forms increase. In particular, in SH-SY5Y/APP- cells, p-ERK1/2, p-CaMKII, and p-CREB decrease by 59.65 ± 14.95% (*p* < 0.001), 62.68 ± 14.71% (*p* < 0.01) and 40.58 ± 11.50% (*p* < 0.01), respectively, while p-CaMKIV levels increase by 223.27 ± 121.61% (*p* < 0.01), compared to SH-SY5Y cells. Consequently, the ratios of un-phosphorylated/phosphorylated forms are lower in SH-SY5Y/APP- cells for ERK1/2 (75.56 ± 7.39%, *p* < 0.001), CaMKII (35.54 ± 25.19%, *p* < 0.05) and CREB (65.00 ± 14.89%, *p* < 0.01), whereas the p-CaMKIV/CaMKIV ratio is approximately 8-fold higher (*p* < 0.05), compared to the corresponding ratio in SH-SY5Y cells. Overall, these data indicate that APP downregulation diminishes the basal activation status of critical synaptic signaling components, with the exception of CaMKIV.

### 2.3. Effects of APP Downregulation on ER Proteins

**Figure 4:**
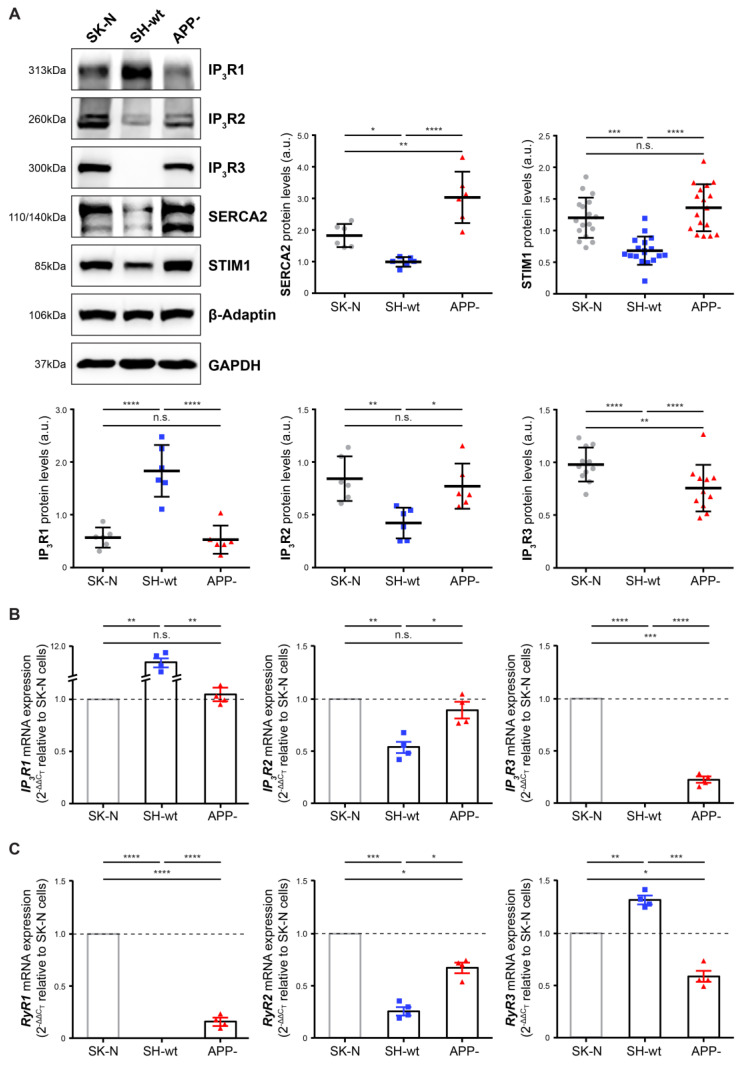
APP downregulation effects on ER membrane proteins. (**A**) Representative immunoblots and quantification of IP_3_R1-3 and STIM1 levels in total protein extracts (50 μg/lane) from SH-SY5Y (SH-wt), SH-SY5Y/APP- (APP-), and SK-N-SH (SK-N) cells. GAPDH or β-Adaptin was used as loading controls for normalization. The molecular weights of the proteins are depicted on the left of each blot image. Data are presented as arbitrary units (a.u.) of the mean ± S.D. (*n* = 6–17). Dots represent individual data points. Statistical significance was evaluated by one-way ANOVA followed by Tukey’s multiple comparison tests (* *p* < 0.05, ** *p* < 0.01, *** *p* < 0.001). (**B**,**C**) Endogenous expression levels of *IP_3_R1-3* (**A**) and *RyR1-3* (**B**) mRNAs in SK-N, SH-wt and APP- cells, evaluated using RT-qPCR analysis, using *GAPDH* mRNA for normalization. Data show the mean ± S.D. from four biological replicates (2^-ΔCT^ relative to SK-N cells, indicated with a dashed line). Dots represent individual data points. Statistical significance was evaluated by one-way ANOVA followed by Tukey’s multiple comparison tests (* *p* < 0.05, ** *p* < 0.01, *** *p* < 0.001, **** *p* < 0.0001). IP_3_R, Inositol 1,4,5-triphosphate receptor; RyR, Ryanodine receptor; SERCA2, Sarco/endoplasmic reticulum Ca^2+^-ATPase; GAPDH, Glyceraldehyde 3-phosphate dehydrogenase.

Next, we focused on the effects of APP downregulation on the ER membrane resident proteins STIM1, which is a specific sensor of the ER Ca^2+^ content, and SERCA2, which pumps Ca^2+^ into the ER lumen. Moreover, the expression of inositol 1,4,5-triphophate (IP_3_R1-3) and ryanodine (RyR1-3) receptors which mediate intracellular Ca^2+^ signaling, was also evaluated. For the detection of SERCA2, we used a primary antibody that recognizes two bands at approximately 110 and 140kDa, both of which were taken into consideration for quantification. Western blot analysis revealed that APP downregulation leads to an increase in STIM1 (by 80.24 ± 50,30%, *p* < 0.001), SERCA2 (by 201.64 ± 81.12%, *p* < 0.001), and IP_3_R2 (by 83.01 ± 50.97%, *p* < 0.001) protein levels, compared to SH-SY5Y cells (Figure 4A). Strikingly, although endogenous levels of IP_3_R3 are undetectable in SH-SY5Y cells, SH-SY5Y/APP- cells display a considerable induction of IP_3_R3 expression, yet lower when compared to SK-N-SH cells (Figure 4A). Conversely, IP_3_R1 levels in SH-SY5Y/APP- cells are decreased by 72.86 ± 10,40% (*p* < 0.001) compared to SH-SY5Y. 

We also tried to evaluate RyR1-3 protein levels by western blot analysis. However, our efforts were unsuccessful, probably due to their moderate abundance in the three cell lines and to technical difficulties related to their very high molecular weight (565 kDa). To circumvent this, we performed RT-qPCR analysis in total RNA extracts, using *IP_3_R1-3* and *RyR1-3* mRNA-specific primers, and data obtained were quantified using the 2-^ΔΔC^_T_ method relative to SK-N-SH cells, with *GAPDH* mRNA serving as a normalization standard. Noticeably, the results obtained for *IP_3_R* mRNAs coincide with the data acquired by western blot analysis; i.e., a decrease in *IP_3_R1* [86.18 ± 2.45% (*p* < 0.01)] and an increase in *IP_3_R2* [(by 49.75 ± 18.43% (*p* < 0.05)] mRNA levels, compared to SH-SY5Y cells. Interestingly, as was the case with protein levels, *IP_3_R3* mRNA was detected in SH-SY5Y/APP- cells but not in SH-SY5Y cells (Figure 4B). RT-qPCR analysis using *RyR1-3* mRNA-specific primers revealed that APP downregulation leads to the upregulation of *RyR2* mRNA levels [up to 268.61 ± 77.27% (*p* < 0.05)], whereas *RyR3* mRNA levels decrease by 55.59 ± 2.93% (*p* < 0.001) (Figure 4C). Interestingly, similar to *IP_3_R3* mRNA expression, *RyR1* mRNA is detected in SH-SY5Y/APP- cells but not in SH-SY5Y cells. However, compared to SK-N-SH cells, the levels of *RyR1* mRNA in SH-SY5Y/APP- cells are decreased by 85.23 ± 4.03% (*p* < 0.001). Collectively, APP downregulation results in a significant increase in the endogenous levels of four out of six subunits of ER Ca^2+^ channels and of STIM1 and SERCA2 (Table 1). 

### 2.4. Wortmannin Reveals a Specific Signaling Pattern in SH-SY5Y/APP- Cells

**Figure 5:**
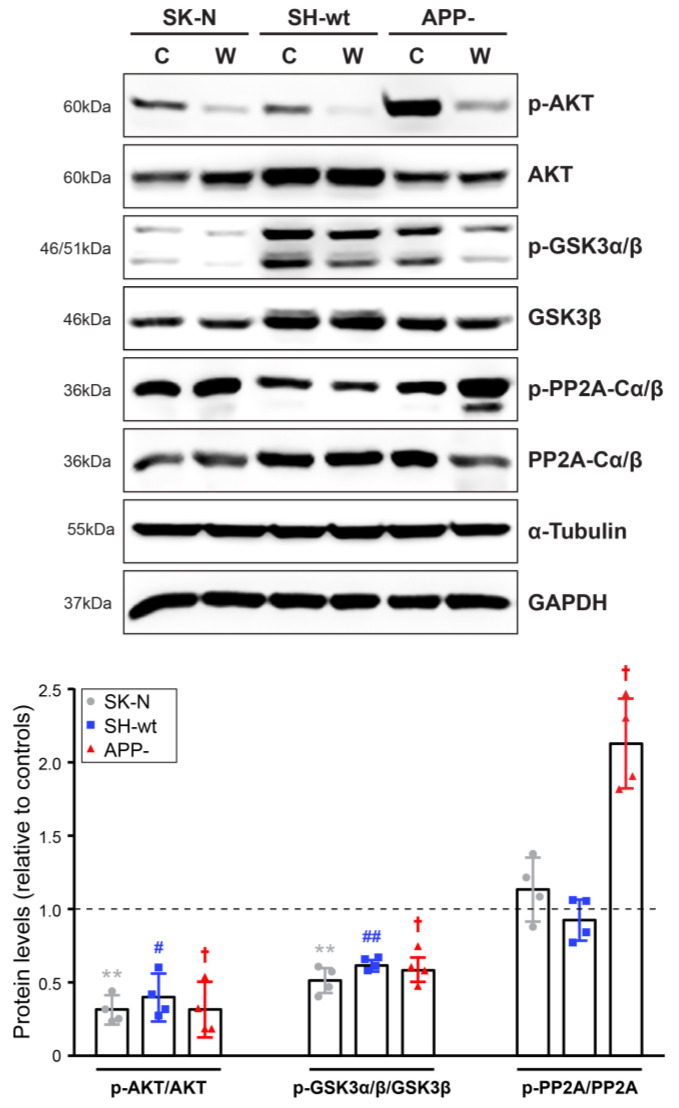
Inhibition of PI3K-AKT by wortmannin deactivates PP2A in SH-SY5Y/APP- cells. Representative immunoblots and quantification of the levels of AKT, GSK3α/β, PP2A-Cα/β and their phosphorylated forms in total protein extracts (40 μg/lane) from SH-SY5Y (SH-wt), SH-SY5Y/APP- (APP-), and SK-N-SH (SK-N) cells after treatment with 100 nM wortmannin (W) for 2 h. GAPDH or α-Tubulin were used as loading controls for normalization. The molecular weights of the proteins are depicted on the left of each blot image. Data are presented as arbitrary units (a.u.) of the mean ± S.D. ratio of the pixel intensity of the phosphorylated bands to the intensity of AKT, GSK3α/β and PP2A-Cα/β total levels in treated cells (W), relative to their respective untreated control cells (C, set at 1, indicated with a dashed line). Dots represent individual data points from four biological replicates. Statistical significance was evaluated by one-way ANOVA followed by Dunnett’s multiple comparison test. “*” is used to depict statistically significant differences between control and treated SK-N cells (** *p* < 0.01), # between control and treated SH-wt cells (# *p* < 0.05, ## *p* < 0.01), while † shows statistically significant differences between control and treated APP- cells († *p* < 0.05). AKT, Alternative appellation for Protein kinase B; GSK3, Glycogen synthase kinase-3; PP2A, Protein phosphatase 2A-C alpha/beta; GAPDH, Glyceraldehyde 3-phosphate dehydrogenase.

**Figure 6:**
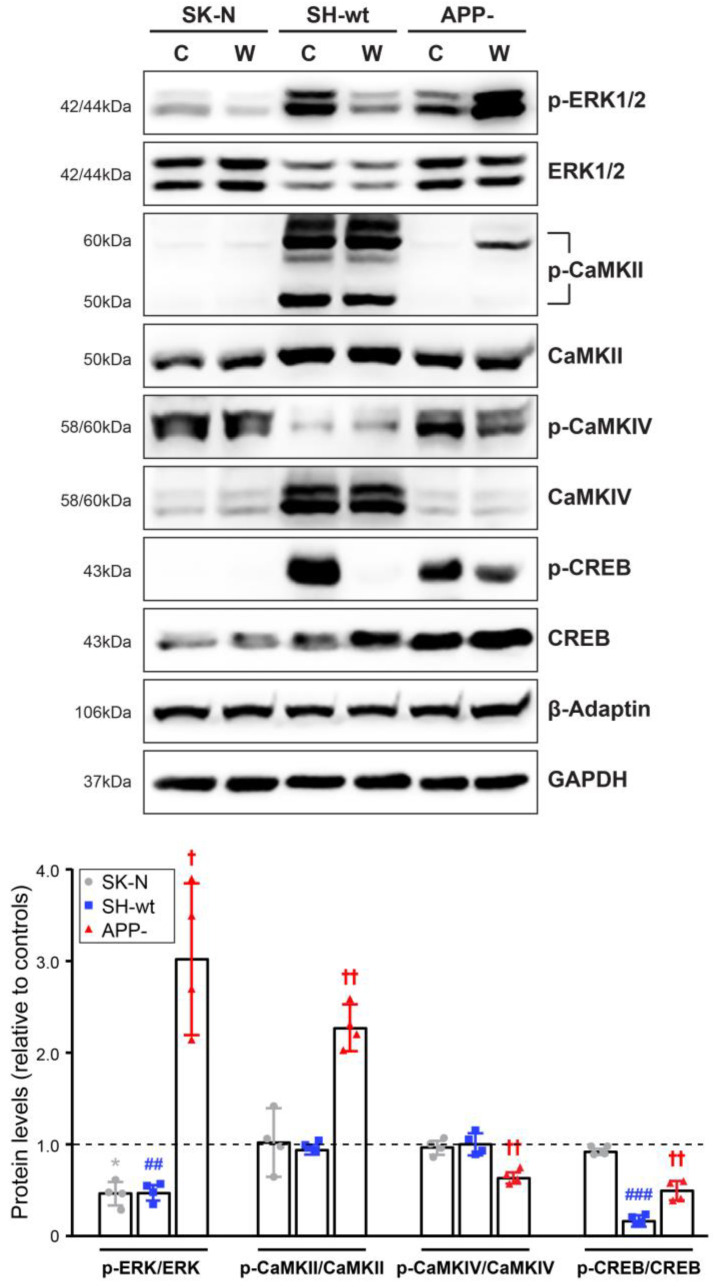
Inhibition of p-AKT by wortmannin activates ERK1/2 and CaMKII in SH-SY5Y/APP- cells. Representative immunoblots and quantification of the levels of ERK1/2, CaMKII, CaMKIV, CREB, and their phosphorylated forms in total protein extracts (40 μg/lane) from SH-SY5Y (SH-wt), SH-SY5Y/APP- (APP-), and SK-N-SH (SK-N) cells, after treatment with 100 nM wortmannin (W) for 2 h. GAPDH or β-Adaptin was used as loading controls for normalization. The molecular weights of the proteins are depicted on the left of each blot image. Data are presented as arbitrary units (a.u.) of the mean ± S.D. ratio of the pixel intensity of the phosphorylated bands to the intensity of ERK1/2, CaMKII, CaMKIV, CREB total levels in treated cells (W) relative to their respective untreated-control cells (C, set at 1, indicated with a dashed line). Dots represent individual data points from four biological replicates. Statistical significance was evaluated by one-way ANOVA followed by Dunnett’s multiple comparison test. “*” is used to depict statistically significant differences between control and treated SK-N cells (* *p* < 0.05), # between control and treated SH-wt cells (## *p* < 0.01, ### *p* < 0.001), while † shows statistically significant differences between control and treated APP- cells († *p* < 0.05, †† *p* < 0.01). ERK1/2, extracellular signal-regulated kinases 1/2; CAMKII, Ca^2+^/calmodulin dependent kinase II; CAMKIV, Ca^2+^/calmodulin dependent kinase IV; CREB, cyclic AMP response element binding protein; GAPDH, Glyceraldehyde 3-phosphate dehydrogenase.

One of the most striking alterations detected in SH-SY5Y/APP- cells is the paradoxically high levels of p-AKT (see Figure 2). Since AKT is considered to function upstream of various interconnected signaling pathways, we next examined whether the inhibition of AKT activation would cause any differential effects on SH-SY5Y/APP- cells. To this end, we used wortmannin, a specific inhibitor of PI3K, which binds to its catalytic domain and prevents the phosphorylation of AKT. By using the same rationale as in Section 2.2, the results are grouped in Figure 5 and Figure 6.

Western blot analysis showed that incubation of cells with 100 nM wortmannin for 2 h leads to a dramatic decrease in p-AKT levels (expressed as p-AKT/AKT ratio) in all cell lines, compared to respective untreated samples [reduction in SK-N-SH: 67.81 ± 9.32% (*p* < 0.01), SH-SY5Y: 58.42 ± 16.07% (*p* < 0.05), SH-SY5Y/APP-: 66.98 ± 19.03% (*p* < 0.05)]. These results indicate that p-PI3K effectively phosphorylates AKT (Figure 5) regardless of the level of APP expression. Furthermore, p-GSK3α/β levels (shown here as p- GSK3α/β/GSK3β ratio) are attenuated in the presence of wortmannin [reduction in SK-N-SH: 45.01 ± 8.29% (*p* < 0.01), SH-SY5Y: 34.34 ± 3.75% (*p* < 0.01), SH-SY5Y/APP-: 37.36 ± 8.86% (*p* < 0.05)]. Surprisingly, p-PP2A-Cα/β levels are altered in an unconventional way, making it not entirely clear whether this is exclusively caused by AKT inhibition. In particular, the p-PP2A-Cα/β/PP2A-Cα/β ratio is increased by 112.69 ± 30.46% (*p* < 0.05) in SH-SY5Y/APP- treated cells compared to their untreated control cells, whereas it remains unaffected in SH-SY5Y or SK-N-SH cells. In SH-SY5Y/APP- cells, therefore, it seems that wortmannin potentiates the phosphorylation of PP2A-Cα/β, but this increase cannot compensate for PI3K inhibition. 

Regarding the second group of signaling proteins examined, our results show that PI3K inhibition by wortmannin differentially affects SH-SY5Y/APP-, compared to SH-SY5Y or SK-N-SH cells (Figure 6). In detail, SH-SY5Y/APP- treated cells display a significant increase in the p-ERK/ERK ratio (by 200.88 ± 81.66%, *p* < 0.05) compared to the corresponding untreated cells, whereas this ratio is reduced by 51.01 ± 12.22% (*p* < 0.05) and 50.97 ± 7.97 (*p* < 0.01) in SK-N-SH and SH-SY5Y treated cells, respectively. Similarly, the p-CaMKII/CaMKII ratio is increased by 2.28-folds (*p* < 0.01) in SH-SY5Y/APP- treated cells, whereas it remains unaffected in SK-N-SH and SH-SY5Y treated cells, compared to the respective untreated controls. In contrast, in the presence of wortmannin, p-CaMKIV/CaMKIV ratio is reduced by 36.85 ± 5.33% (*p* < 0.01) only in SH-SY5Y/APP- cells, while the p-CREB/CREB ratio is reduced by 82.98 ± 6.29% (*p* < 0.001) and 50.10 ± 10.49% (*p* < 0.01) in SH-SY5Y and SH-SY5Y/APP- treated cells, respectively. 

Overall, among the cell lines examined, SH-SY5Y/APP- cells respond differently to PI3K inhibition by wortmannin with p-PP2A-Cα/β, p-ERK1/2, p-CaMKII and, to some extent, p-CREB, displaying increased levels.

### 2.5. Responses of SH-SY5Y/APP- Cells in Different Physiological and Pathological Settings

#### 2.5.1. Activation of AKT

**Figure 7:**
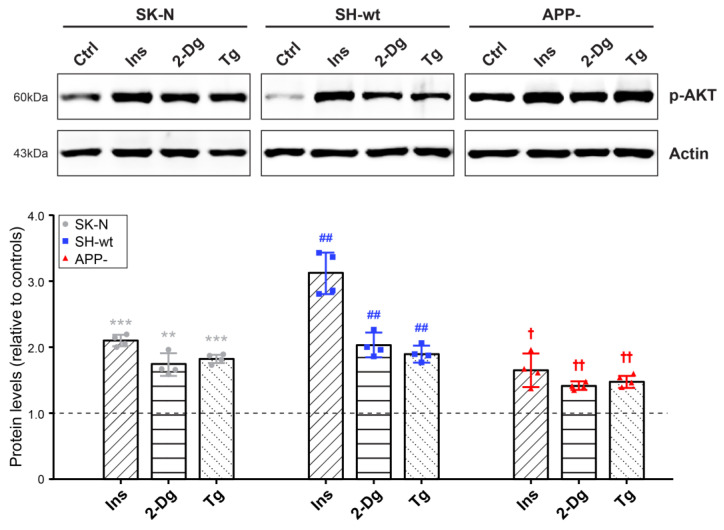
SH-SY5Y/APP- cells display a reduced response to physiological stimuli. Representative immunoblots and quantification of the levels of p-AKT in total protein extracts (30 μg/lane) from SK-N-SH (SK-N), SH-SY5Y (SH-wt), and SH-SY5Y/APP- (APP-) cells after treatment with 3.5 μg/mL insulin (Ins), 1 mM 2-Deoxy-d-glucose (2-Dg) or 250 nM thapsigargin (Tg) for 2 h. Actin was used as a loading control for normalization. The molecular weights of the proteins are depicted on the left of each blot image. Data are presented as arbitrary units (a.u.) of the mean ± S.D. pixel intensity of p-AKT bands in treated cells relative to their respective untreated-control cells (Ctrl, set at 1, indicated with a dashed line) from four biological replicates. Dots represent individual data points from four biological replicates. Statistical significance was evaluated by one-way ANOVA followed by Dunnett’s multiple comparison test. “*” is used to depict statistically significant differences between control and treated SK-N cells (* *p* < 0.05, ** *p* < 0.01, *** *p* < 0.001), # between control and treated SH-wt cells (## *p* < 0.01, ### *p* < 0.001), while † shows statistically significant differences between control and treated APP- cells († *p* < 0.05, †† *p* < 0.01). AKT, Alternative appellation for Protein kinase B.

Considering that AKT levels are significantly altered in SH-SY5Y/APP- cells, we examined whether APP downregulation leads to distinct responses to several stimuli such as insulin, 2-Deoxy-d-glucose (2-Dg), and thapsigargin (Tg) previously shown to activate AKT [28]. Our results showed that incubation of the three cell types with 3.5 μg/mL insulin for 2 h results in a statistically significant increase in p-AKT levels in all cases, but in SH-SY5Y/APP-treated cells, this response is lower [increase by 65.36 ± 25.05% (*p* < 0.05)] than that elicited in SH-SY5Y [increase by 212.33 ± 32.15% (*p* < 0.01)] and SK-N-SH [increase by 110.04 ± 8.39% (*p* < 0.001)] cells (Figure 7). Next, we examined the AKT responses during stress conditions, exemplified by the use of 2-Dg, which mimics glucose deprivation, and Tg, which induces ER Ca^2+^ depletion. We found that APP downregulation leads to a lower AKT activation induced by 2-Dg and Tg [increase by 41.69 ± 7.06% (*p* < 0.01) and 50.01 ± 8.85% (*p* < 0.01), respectively], compared to SH-SY5Y [increase by 103.37 ± 15.44% (*p* < 0.01) and 89.27 ± 10.98% (*p* < 0.01)] and SK-N-SH cells [increase by 74.60 ± 17.41% (*p* < 0.01) and 81.60 ± 6.41% (*p* < 0.001)], which display a stronger response to these stimuli.

#### 2.5.2. Ca^2+^ Mobilization through IP_3_Rs

**Figure 8:**
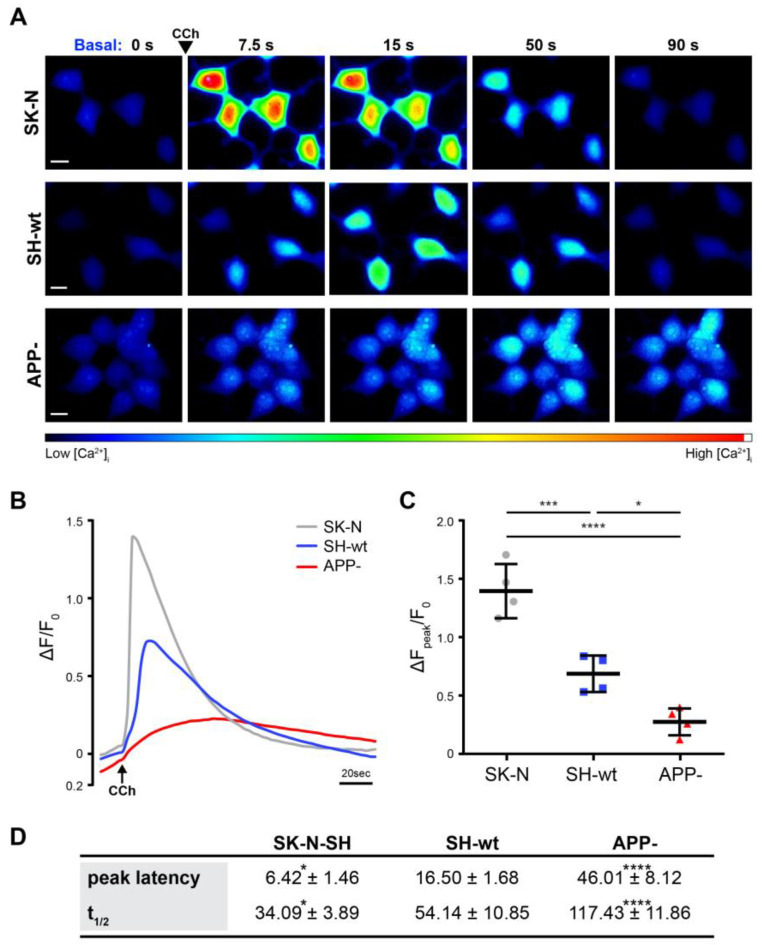
Carbachol-induced ER Ca^2+^ release is attenuated in SH-SY5Y/APP- cells. (**A**). Representative fluorescence images of SK-N-SH (SK-N), SH-SY5Y (SH-wt), and SH-SY5Y/APP- (APP-) cells loaded with Fluo-4. Carbachol (CCh, 1 mM) was added in extracellular Ca^2+^-free conditions, and fluorescence intensity was monitored over time. Scale bars: 10 μm. (**B**) Mean Ca^2+^ amplitude curves where ΔF/F_0_ represents changes in the fluorescence intensity over baseline levels over time. (**C**) Comparison of the mean peak amplitudes of the fluorescence signals expressed as ΔF_peak_/F_0_ (a.u.). (**D**) Comparison of CCh-induced Ca^2+^ kinetics (peak latency and the half-time decay (t_1/2_) of the responses, in seconds (s)) between the three cell lines. All data show the mean ± S.D. of four independent experiments. Dots represent individual data points. Statistical significance was evaluated by one-way ANOVA followed by Tukey’s multiple comparison tests (* *p* < 0.05, *** *p* < 0.001, **** *p* < 0.0001).

Finally, we monitored the ER Ca^2+^ release induced by carbachol (CCh), a muscarinic agonist that causes phospholipase-mediated IP_3_ formation and subsequent Ca^2+^ release from ER stores through IP_3_Rs [29,30,31]. Ca^2+^ release can subsequently activate RyRs and broaden the response via the Ca^2+^-induced Ca^2+^ release pathway [32]. Ca^2+^ measurements were carried out in the absence of extracellular Ca^2^ to assess the effects of CCh on Ca^2+^ release from the ER. Based on IP_3_R and RyR mRNA and protein levels (Figure 4), we would expect that the response of SH-SY5Y/APP- cells would be comparable to that of SK-N-SH cells. Strikingly, our results showed that despite the increased levels of IP_3_R2, IP_3_R3, RyR1, and RyR2, SH-SY5Y/APP- cells generate a smaller rise in Ca^2+^ release from the ER (Figure 8). In particular, quantification of fluorescence intensities (ΔF/F_0_), corresponding to intracellular Ca^2+^ levels upon acute addition of 1 mM CCh, revealed that the increase in the mean Ca^2+^ amplitude is significantly lower in SH-SY5Y/APP- cells than SH-SY5Y (*p* < 0.05) and in SK-N-SH cells (*p* < 0.001) (Figure 8B,C). In addition, SH-SY5Y/APP-cells exhibited a delayed response (peak latency) to CCh by 46.01 ± 8.12 sec (*p* < 0.001), and the half-time decay time (*t_1/2_*) of their response to basal levels lasted longer and reached 117.43 ± 11.86 sec, (*p* < 0.001) (Figure 8A,D). This prolonged activation, likely due to IP_3_R redundancies, fails to restore ER Ca^2+^ release upon CCh stimulation. Noticeably, Ca^2+^ imaging measurements in Ca^2+^ containing medium, prior to stimulation with an agonist, showed that the basal levels of [Ca^2+^]_i_ in SH-SY5Y/APP- cells (164.19 ± 24.83 nM) are increased by 52.37 ± 23.04% (*p* < 0.001), compared to SH-SY5Y (107.76 ± 14,95 nM) and SK-N-SH cells (98.07 ± 9.60 nM).

## 3. Discussion

**Figure 9:**
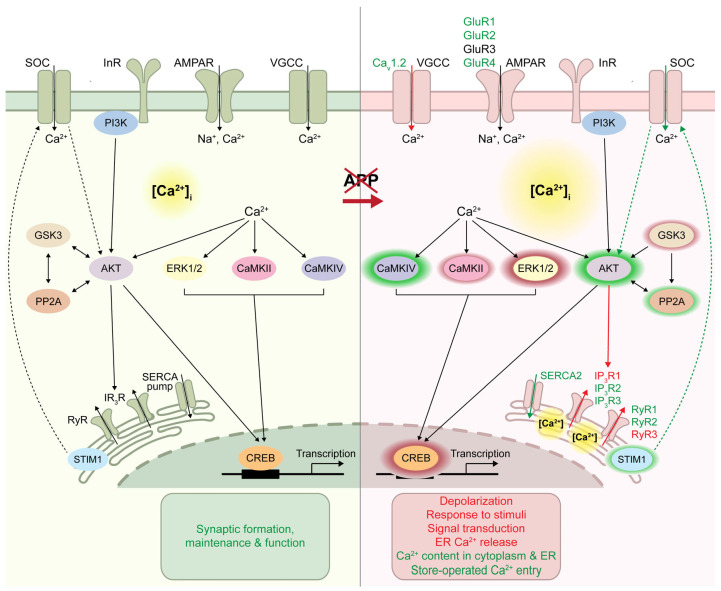
Schematic representation of key signal transduction players that govern Ca^2+^ signaling during synaptic function and are mainly affected by APP downregulation. This illustration combines our findings with data available from the literature discussed in the text. The shaded halos shown on the right side (APP knockdown) of the image were used to surround proteins that are either upregulated (green) or downregulated (red), as evidenced by the phosphorylated/total ratio. Their size is proportional to the extent of the upregulation or downregulation. Arrows represent increases (green) or decreases (red) in the activity of the respective channels or pathways.

We assessed the expression pattern of key signal transduction proteins that mediate Ca^2+^ mobilization during synaptic function, and we found that the vast majority of them are significantly altered in SH-SY5Y/APP- cells compared to SH-SY5Y (Table 1, Figure 9). Specifically, while the levels of GluR3, PP2A-Cα/β, and p-PI3K remained unaffected, the levels of PI3K, p-AKT, p-CaMKIV, STIM1, SERCA2, IP_3_R3, and CREB were upregulated, and those of p-GSK3α/β, p-ERK1/2, p-CaMKII, IP_3_R1, and CaMKIV was downregulated. Interestingly, the expression profile of the proteins examined did not differ significantly between SH-SY5Y/APP- and moderately expressing APP SK-N-SH cells, with the exception of p-AKT, CREB/p-CREB, and IP_3_R3. This observation also applies to *IP_3_R3* and *RyR1* mRNAs, whose levels were not detected in SH-SY5Y cells. The above finding may reflect the role of APP in neuronal differentiation, as the SH-SY5Y cell line is a clone derived from SK-N-SH cells and can differentiate into a more mature neuronal phenotype [33]. Nevertheless, the possibility that the differences in the levels of these proteins observed between SK-N-SH and SH-SY5Y cells could be due to effects other than APP expression levels cannot be excluded.

In the mammalian brain, AMPARs are tetrameric membrane complexes consisting of GluR1-4 subunits in various combinations that mediate the fast excitatory synaptic glutamate transmission. GluR1, GluR3, and GluR4, as well as the unedited form of GluR2, are Ca^2+^-permeable and, as a consequence, are predisposed to excitotoxicity [34]. In various models of AD, trafficking, localization, and function of GluR subunits have all been shown to be dysregulated [35], and, GluR1, by far the most extensively studied GluR subunit, is associated with both synaptic plasticity and neurodegeneration [36]. Although our efforts to detect GluR1 in the cell lines used were unsuccessful, Martinsson et al. [37] have recently shown that APP knockdown in primary neurons results in increased levels of GluR1. Our results clearly showed that the expression of GluR2 and GluR4 subunits was also increased in the absence of APP. It seems, therefore, that APP downregulation associates well with increased expression of several plasma membrane ion channels, and remarkably, this tends to be opposite to the reduction in GluR1 seen in *APP* mutant neurons [38]. Altogether, these data indicate that full-length APP accounts for the normal composition and function of synapses and the hyperexcitability observed in models of AD [39]. Thus, it would be of great interest to evaluate in the future the expression and activity of other plasma membrane ions channels, such as NMDARs, GABARs, VGCCs, and SOCs, that mediate Ca^2+^ signaling and might be altered in the absence of APP. 

Ca^2+^ channels residing at the ER membrane are also profoundly affected in the absence of APP (Figure 4), pointing to an ER Ca^2+^ dysregulation frequently linked to neurodegeneration [40]. Alterations of both IP_3_Rs and RyRs expression and function were previously reported in several models of AD [41,42]. Here we show a significant decrease in the expression of IP_3_R1, the predominant isoform in neurons [43]. More strikingly, although endogenous levels of IP_3_R3 were undetectable in SH-SY5Y cells, SH-SY5Y/APP- cells displayed a considerable induction of IP_3_R3 expression. Moreover, we found that the expression of both STIM1 and SERCA2 is increased in SH-SY5Y/APP- cells (Figure 4). In the brain, STIM1, apart from binding to SOCs, regulates Ca^2+^ entry mediated by glutamate receptors and VGCCs and hence, contributes to excitability [44]. It has been previously shown that STIM1 protein expression levels decreased during neurodegeneration and knock-out of *STIM1* gene expression, using CRISPR/Cas9-mediated genome editing in SH-SY5Y cells, diminished IP_3_R3 protein levels [45]. Our results corroborate this finding, as protein levels of both STIM1 and IP_3_R3 were increased in SH-SY5Y APP- cells, and probably this applies also to IP_3_R2. Additional experiments are required to elucidate the mechanisms by which STIM1 may regulate the expression of IP_3_R3 and IP_3_R2, while their interrelationship may reflect a novel regulatory module of Ca^2+^ efflux from the ER. 

Given that the majority of GluR, IP_3_R, and RyR subunits are increased upon APP downregulation (Table 1), Ca^2+^ homeostasis and signaling are expected to be also affected. To examine this hypothesis, we triggered Ca^2+^ mobilization from the ER using CCh in Ca^2+^-free conditions, and surprisingly, we found that ER Ca^2+^ release is decreased but prolonged (Figure 8). Moreover, cytosolic Ca^2+^ levels are significantly elevated in SH-SY5Y/APP- cells, compared to both SH-SY5Y and SK-N-SH, an effect that could restrict CCh-induce ER Ca^2+^ release. These findings agree with previously reported results on mouse embryonic fibroblasts where these disturbances could be reversed by transfection of constructs expressing the APP intracellular domain (AICD) [18]. We also observed a partial reversal of STIM1 levels upon transient transfection of SH-SY5Y/APP- cells with an AICD-carrying plasmid (data not shown). However, a more detailed analysis is required to establish whether AICD exerts negative regulatory effects on the transcription of the numerous proteins that are upregulated in the absence of APP. 

Prolonged discharging of ER Ca^2+^ stores was also detected in APP-deficient T84 human colon carcinoma cell line, using cyclopiazonic acid to inhibit the SERCA. Under these conditions, it was shown that, although STIM1 levels were unaffected, its translocation to its plasma membrane partner ORAI1 was delayed, and ER Ca^2+^ content remained increased [46]. In support of this observation, we have previously demonstrated that the downregulation of APP enhances the ER Ca^2+^ content and the sensitivity of SOCs to the specific inhibitor SKF-96365, leading to a faster inhibition of Ca^2+^ entry [19]. We hypothesized that, in the absence of APP, a structural modulation exposes the susceptibility to the inhibition site of the channel. However, the possibility of downregulated SOCs expression, as it was shown in cortical astrocytes from APP knockout mice [47], cannot be excluded. Furthermore, the higher amount of ER Ca^2+^ content could be attributed to the observed enhanced expression of SERCA2 and/or altered ER Ca^2+^ channel function, despite the higher expression of the latter. Considering that all three isoforms of IP_3_R can be phosphorylated and thus inactivated by AKT [48], the high p-AKT levels detected in the absence of APP could account for this effect. 

Ca^2+^ mobilization is immediately sensed by calmodulin and triggers the activation of CaMKII, CaMKIV, and ERK1/2, ultimately leading to the phosphorylation and subsequent activation of CREB in the nucleus, conferring neuroprotection [20,21,22,49]. These Ca^2+^ signaling molecules may represent regulatory targets for APP. We found that the downregulation of APP caused a statistically significant decrease in p-CaMKII, p-ERK1/2 and p-CREB levels, but p-CaMKIV levels were particularly enhanced, despite low levels of the respective total proteins (Figure 3). ERK is a widely expressed serine-threonine protein kinase, reported to play an essential role in neuronal survival and synaptic plasticity [50], and enhanced phosphorylation of ERK1/2 is associated with early tau deposition in AD [51]. CaMKII is the most important Ca^2+^ sensor transducing glutamatergic activation into synaptic plasticity through structural adaptations at the synapse during learning processes [52]. CaMKII has also been implicated in tau [53] and even APP phosphorylation [54], but the nature of CaMKII dysregulation during neurodegeneration is still unclear. In contrast, it has been shown that soluble Aβ (1–42) increased the phosphorylation of CaMKIV due to the increase in basal Ca^2+^ concentration [55]. Remarkably, at low Ca^2+^ levels, PP2A binds to and dephosphorylates CaMKIV, hence affecting neuroprotection [56,57]. Our findings show that phosphorylation, and thus inactivation, of PP2A, is increased in the absence of APP (Figure 2), indicating a possible mode of CaMKIV phosphorylation. It is worth noting that p-CREB levels were diminished in SH-SY5Y/APP- cells while retaining a statistically significant difference compared to SK-N-SH cells (Figure 3). However, whether this difference can be attributed to p-CaMKIV and whether it occurs at the nucleus remain to be elucidated. 

One of the most remarkable findings of this study is the increased p-AKT levels upon APP downregulation. The PI3K-AKT pathway constitutes the main survival pathway regulating many cellular functions, including inhibition of Aβ-induced neurotoxicity [58,59]. We have previously demonstrated that glucose deprivation, although cytotoxic, is accompanied by AKT activation and Ca^2+^ entry [28]. In support of this finding, it was reported that specific Ca^2+^ channels in lipid rafts comprise important sites linking Ca^2+^ entry to AKT directly signaling [60], a phenomenon that could go with hyperactivation of SOC. Based on the above, we hypothesized that Ca^2+^ entry activates PI3K/AKT, which in turn is engaged to support Ca^2+^ channel trafficking and hence, is not accessible to downstream pathways. Taking into consideration that the absence of APP was found to render SOCs more vulnerable to inhibition [19], we could also hypothesize that APP affects the structural conformation of these channels, resulting in the adjustment of their function. Thus, it may be possible that in the absence of APP, facilitated Ca^2+^ entry could account for the high levels of p-AKT. 

Activation of AKT inhibits GSK3 kinase by phosphorylation, and therefore, we would expect to detect enhanced p-GSK3 levels in SH-SY5Y/APP- cells. Interestingly, we found the opposite, i.e., in SH-SY5Y/APP- cells, p-GSK3 levels were almost 50% of the levels found in SH-SY5Y, expressing low levels of p-AKT. GSK3 has been shown to phosphorylate several upstream and downstream proteins [27]. Accordingly, it is possible that, in the absence of APP, GSK phosphorylates AKT and PP2A, which may result in tau hyperphosphorylation. This, in turn, leads to the formation of neurofibrillary tangles, and hence, both of these proteins are comprehensively considered in the neuropathology of AD [61,62]. Based on our findings, we hypothesize that APP may shape the balance between p-GSK and p-PP2A because, in all cases, when p-GSK is increased, p-PP2A is decreased and vice versa (Figure 2), and their activity is therefore mutually exclusive. This is also evident in the case of PI3K/AKT pathway inhibition by wortmannin (Figure 5), where p-GSK levels were significantly reduced while p-PP2A levels were impressively augmented in SH-SY5Y/APP- cells. This finding also indicates that PP2A phosphorylation is mediated predominantly by GSK and not by p-AKT. Furthermore, p-PP2A, p-ERK1/2, and p-CaMKII levels were also significantly enhanced in SH-SY5Y/APP- cells in the presence of wortmannin (Figure 6) and their possible interconnection would be of great interest to investigate in order to understand the mechanisms of APP contribution to the formation of a broad network of signaling pathways.

## 4. Materials and Methods

### 4.1. Cell Culture and Treatments

SK-N-SH and SH-SY5Y human neuroblastoma cells were cultured in 100 mm plates (Greiner bio-one, Cellstar, Kremsmuenster, Austria) in high-glucose Dulbecco’s Modified Eagle Medium (DMEM) (Biosera, Nuaille, France), supplemented with 10% fetal bovine serum (Biosera) and 1% of penicillin/streptomycin (Biosera), and maintained at 37 °C in a humidified 5% CO_2_ incubator. For APP downregulation, SH-SY5Y cells were infected with the recombinant lentivirus, purchased from Sigma-Adrich (St. Louis, MO, USA), expressing the shRNA targeting the coding region of the APP gene (CGGGCCATCTTTGACCGAAACGAACTCGAGTTCGTTTCGGTCAAAGATGGCTTTTT). After infection, stable clones in which APP695 was downregulated (SH-SY5Y/APP-) were selected using the antibiotic puromycin (Interchim, Montlucon, France) at a concentration of 2 μg/mL. APP downregulation was verified using a western blot.

Wortmannin (100 nM, Sigma-Aldrich) was used as a specific inhibitor of the PI3K/AKT pathway. Insulin (Ins, 3.5 μg/mL; #I9278, Sigma-Aldrich), 2-Deoxy-d-glucose (2-Dg, 1 mM; #D8375, Sigma-Aldrich), and thapsigargin (Tg, 250 nM; #J-62866, Alfa Aesar, Karlsruhe, Germany) were used to analyze their effects on AKT activation in SH-SY5Y/APP- cells. Finally, carbachol (#L-06674, Alfa Aesar) was used to assess Ca^2+^ release from the ER.

### 4.2. Preparation of Total Protein Extracts and Western Blot Analysis

SK-N-SH, SH-SY5Y, or SH-SY5Y/APP- cells were washed twice with phosphate-buffered saline (PBS) and resuspended in ice-cold RIPA lysis buffer containing 50 mM Tris-HCl (pH 7.5), 150 mM NaCl, 1% Triton X-100, 0.1% SDS, and supplemented with 1 x protease (Complete Protease Inhibitor Cocktail Tablets) and protein phosphatase (PhosSTOP Phosphatase Inhibitor Cocktail Tablets) inhibitors (both from Roche, Basel, Switzerland). Cell suspensions were incubated for 30 min on ice and afterward were centrifuged for 30 min at 13,000× *g* rpm at 4 °C. The supernatants were transferred in new tubes and stored at −80 °C until use. Total protein was measured and quantified using Pierce BCA Protein Assay Kit (ThermoFisher Scientific, Waltham, MA, USA).

Equal amounts of whole protein extracts (40–50 μg) were separated using 8–10% SDS polyacrylamide gel electrophoresis (SDS-PAGE) under denaturing conditions and transferred to nitrocellulose membranes (Porablot NCP; Macherey-Nagel, Düren, Germany). Membranes were blocked in blocking solution [5% BSA and 0.1% Tween-20 in Tris-buffered saline (TBS)] for 1 h at room temperature, followed by overnight incubation at 4 °C with the respective primary and secondary antibodies (see Supplementary Table S1), diluted in blocking solution. The immunoreactive bands were visualized with the enhanced chemiluminescence (ECL) method using the Luminata™ Crescendo Western HRP Substrate (Millipore, Billerica, MA, USA) in an 8800 FluorChem Imaging System (Alpha Innotech Corp., San Leandro, CA, USA). The BlueStar Plus Prestained Protein Marker (Nippon Genetics, Tokyo, Japan) was used to confirm the molecular weight of the examined proteins. Biological samples obtained from more than three independent experiments, noted in figure legends, were used for analysis. The intensity of each immunoreactive band was estimated by densitometric quantification using the ImageJ/Fiji software (version 1.53t; https://fiji.sc/; National Institute of Health (NIH), Madison, WI, USA). GAPDH, β-adaptin, α-tubulin, or actin were used as loading controls for normalization.

### 4.3. Total RNA Purification and RT-qPCR Analysis

Total RNA from SK-N-SH, SH-SY5Y-wt, or SH-SY5Y/APP− was extracted using the TRIzol^®^ Reagent according to the manufacturer’s instructions (ThermoFisher). Briefly, cDNA was synthesized from 1 μg total RNA using random hexamer primers according to the M-MLV reverse transcriptase protocol (ThermoFisher). The resulting cDNA was diluted (10x) with nuclease-free water (ThermoFisher) and used for qPCR analysis, which was carried out based on SYBR^®^ Green I DNA binding dye (ThermoFisher). The reaction mixture (20 μL total volume per well) included 9 μL of cDNA (~20 ng), 9.6 μL Kapa SYBR^®^ Fast Universal 2 x qPCR Master Mix (Kapa Biosystems, Roche, Basel, Switzerland), 0.4 μL of 50 x Rox Low passive reference dye (Kapa Biosystems) and primers at a final concentration of 200 nmol/L. qPCR analysis was carried out in 96-well PCR microplates (Applied Biosystems, Forster City, CA, USA) on a 7500 Real-Time PCR System (Applied Biosystems) and the 7500 Real-Time PCR Software v2.3 (Applied Biosystems) was used for quantification of the PCR products. Each sample was tested in triplicate, and data obtained from 4 independent biological samples were analyzed using the 2-^ΔΔC^_T_ method with GAPDH mRNA for standardization. PCR conditions and primer sequences for RT-qPCR were as described previously [63].

### 4.4. Ca^2+^ Imaging

For Ca^2+^ measurements, cells seeded onto 25 mm diameter round glass coverslips were loaded with the Ca^2+^ indicator Fluo-4/AM (2 μΜ) and 100 μΜ DTPA for 30 min at 30 °C in the dark. Cells were then washed three times with Krebs-HEPES buffer (KRH: 135 mM NaCl, 5.9 mM KCl, 1.2 mM MgCl_2_, 11.6 mM HEPES, 11.5 mM Glucose, 1.5 mM CaCl_2_, pH 7.4) and incubated with KRH buffer for an additional 30 min at 25 °C in the dark to allow complete intracellular de-esterification of the dye. In order to measure the response to carbachol in Ca^2+^ free conditions, cells were washed three times with a Ca^2+^ free KRH buffer, in which CaCl_2_ was replaced with 1 mM of the Ca^2+^ chelating agent EGTA. The coverslips were placed and secured in an Attofluor cell chamber (Thermofisher) containing KRH buffer. The metallic chamber was positioned on a 35 mm diameter stage holder of a Zeiss Axio Observer Z1 inverted microscope equipped with a UV source, proper filters, and an AxioCAM HR R3 camera. 

For visualization of Fluo-4, ROIs were drawn for all cells in a specified area, and fluorescence was continuously monitored, with excitation at 450–490 nm bandpass filter and emission at 500–550 nm bandpass filter, on a Carl Zeiss Microscope (Carl Zeiss Microscopy GmbH, Munchen, Germany) equipped with a cooled charge-coupled device (CCD) camera (PTIIC200) (Princeton Instruments, Trenton, NJ, USA). At the end of each experiment, the signals of Fluo4 were calibrated with the sequential addition of 10 μM Ionomycin (Interchim, Montlucon, France), 1% Triton, and 25 mM EGTA to obtain the maximum and minimum fluorescence. Changes in Ca^2+^ were determined using the ZEN Software (version 2.6; Carl Zeiss Microscopy GmbH) for temporal analyses of single cells to express the data as fluorescence ratios as previously described [63]. Briefly, the amplitudes of the Ca^2+^-signals were quantified as ΔF/F_0_ = [(F − F_0_)/F_0_], defined as the change in fluorescence intensity relative to baseline, where F is the fluorescence intensity at any given time and F_0_ is the minimum baseline fluorescence value before stimulation with 1 mM carbachol (CCh). In order to determine any differences in the time-course of the CCh-stimulated increase in [Ca^2+^]_i_, the peak latency (time from baseline to maximal response) and the half-time of decay (t_1/2_ = time from maximal response to half-maximal decay) were also calculated.

### 4.5. Data Analysis

Protein levels were normalized using GAPDH, β-adaptin, α-tubulin, or actin as internal loading controls. All data are presented as mean ± standard deviation (S.D.) of at least 3 independent experiments. In treated samples, the pixel intensity values of the examined proteins were expressed as fold change of control-untreated samples. Depending on the different groups examined, comparisons were carried out using one-way ANOVA, followed by either Tukey’s or Dunnett’s multiple-comparison test. The threshold for statistically significant differences was set to probability values (*p*) less than 0.05. Statistical analysis was performed using the GraphPad Prism software (version 8.0.0; San Diego, CA, USA).

## 5. Conclusions

Herein, we report that the downregulation of APP is accompanied by alterations in the expression of several proteins, all cross-talking with Ca^2+^ signaling, which is important for normal cell function (Figure 9). To our knowledge, many of these alterations are reported for the first time, and their multiplicity places APP in the epicenter of a functional network of plasma and ER membrane components leading to effective signal transduction. APP has been extensively studied as the source of the neurotoxic Aβ peptide characteristic of AD. Chromosomal alterations, such as trisomy of chromosome 21 [64], duplications, and triplications [65,66] of the *APP* gene locus, increase APP expression and lead to AD’s pathology. In addition, overexpression of wild-type human APP in mice causes neuronal loss and cognitive impairment [67]. Reducing the expression of APP could be a good treatment approach if its absence had no impact, given the numerous APP molecular interactions [6]. Thus, a detailed understanding of these interactions will shed light on the complex network of APP-regulated signaling pathways and will improve our understanding of the enigmatic role of APP on cell homeostasis. 

## Figures and Tables

**Table 1:** Comparison of protein expression levels between SH-SY5Y/APP- cells and SH-SY5Y cells. AKT, Alternative appellation for Protein kinase B; CAMKII, Ca^2+^/calmodulin dependent kinase II; CAMKIV, Ca^2+^/calmodulin dependent kinase IV; CREB, cyclic AMP response element binding protein; ERK1/2, extracellular signal-regulated kinases 1/2; GluR, Glutamate receptor subunits; GSK3, glycogen synthase kinase-3; IP_3_R, Inositol 1,4,5-triphosphate receptor; PI3K, Phosphatidylinositol-3 kinase; PP2A-Cα/β, Protein phosphatase 2A-C alpha/beta; RyR, Ryanodine receptor; SERCA2, Sarco/endoplasmic reticulum Ca^2+^-ATPase; STIM1, Stromal interaction molecule 1.

Upregulated		Downregulated		Unmodified
GluR2	PI3K	IP_3_R1	AKT	GluR3
GluR4	ERK1/2	RyR3	GSK3β	p-PI3K
IP_3_R2	CREB		CaMKII	PP2A-Cα/β
IP_3_R3	p-AKT		CaMKIV	
RyR1	p-CaMKIV		p-GSK3α/β	
RyR2	p-PP2A-Cα/β		p-ERK1/2	
SERCA2			p-CaMKII	
STIM1			p-CREB	

## Data Availability

The data presented in this study are available on request from the corresponding author.

## Supplementary Materials

The following are available online at https://www.mdpi.com/article/10.3390/ijms24032302/s1.

## Abbreviations

Aβ, amyloid-β; AD, Alzheimer’s disease; AKT, an alternative appellation for Protein kinase B; AMPAR, α-amino-3-hydroxy-5-methyl-4-isoxazolepropionic acid receptor; APP, β-amyloid precursor protein; CAMKII, Ca^2+^/calmodulin dependent kinase II; CAMKIV, Ca^2+^/calmodulin dependent kinase IV; CCh, carbachol; CREB, cyclic AMP response element binding protein; ER, endoplasmic reticulum; ERK1/2, extracellular signal-regulated kinases 1/2; GAPDH, glyceraldehyde 3-phosphate dehydrogenase; GSK3, glycogen synthase kinase-3; IP_3_R, Inositol 1,4,5-triphosphate receptor; PI3K, Phosphatidylinositol-3 kinase; PP2A, protein phosphatase 2A; RyR, Ryanodine receptor; SERCA2, sarco/endoplasmic reticulum Ca^2+^-ATPase; SOC, Store-operated Ca^2+^ channel; STIM1, Stromal interaction molecule 1; Tg, Thapsigargin.
