# Supplementary figures and images for: Knockdown of Amyloid Precursor Protein Increases Ion Channel Expression and Alters Ca2+ Signaling Pathways
Source: Int J Mol Sci. 2023 Jan 24;24(3):2302. doi: 10.3390/ijms24032302 (PMC9917207; doi:10.3390/ijms24032302)

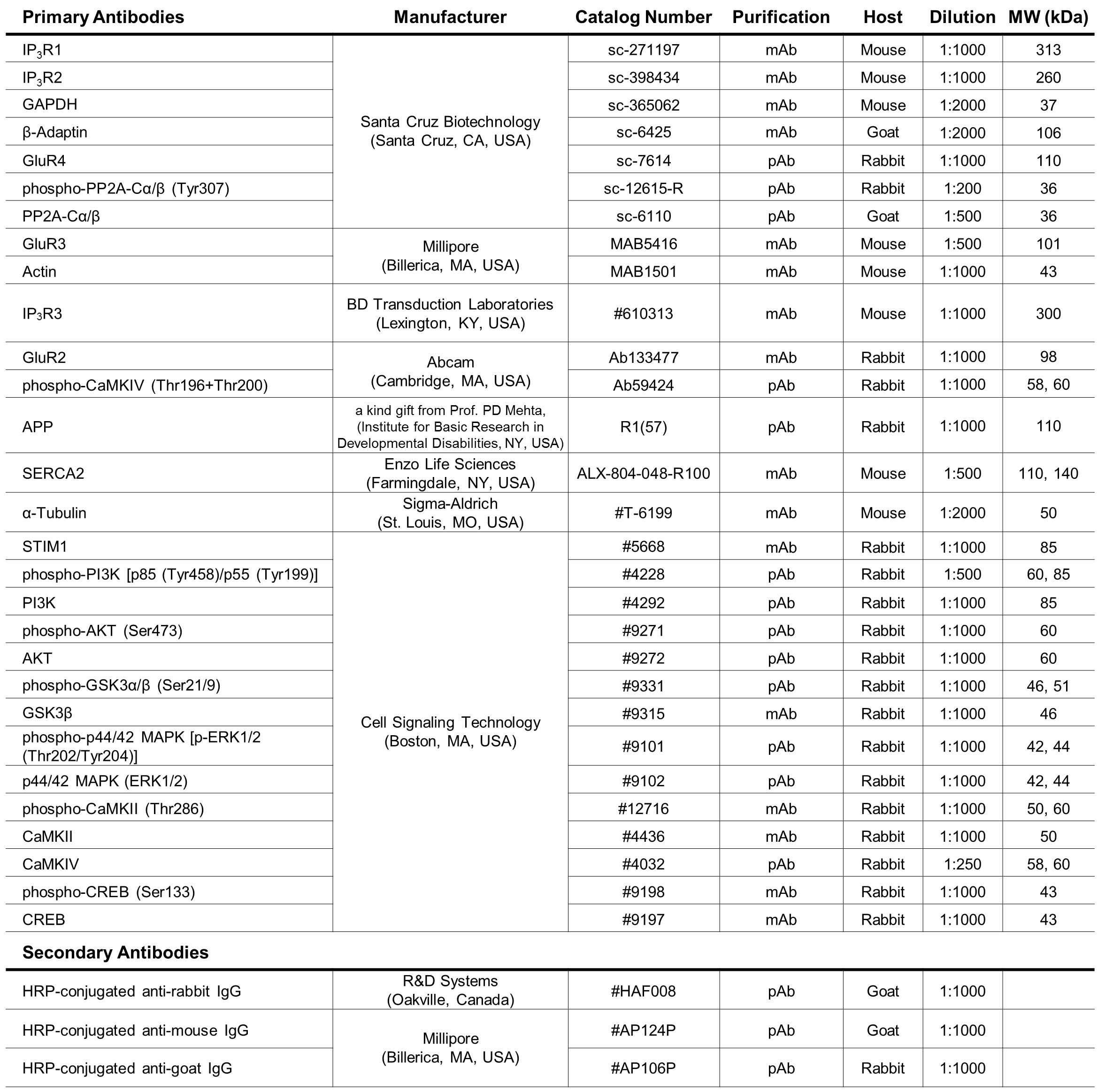

Supplement: Supplementary file 1 [file ijms-24-02302-s001.zip › Table S1.tif]

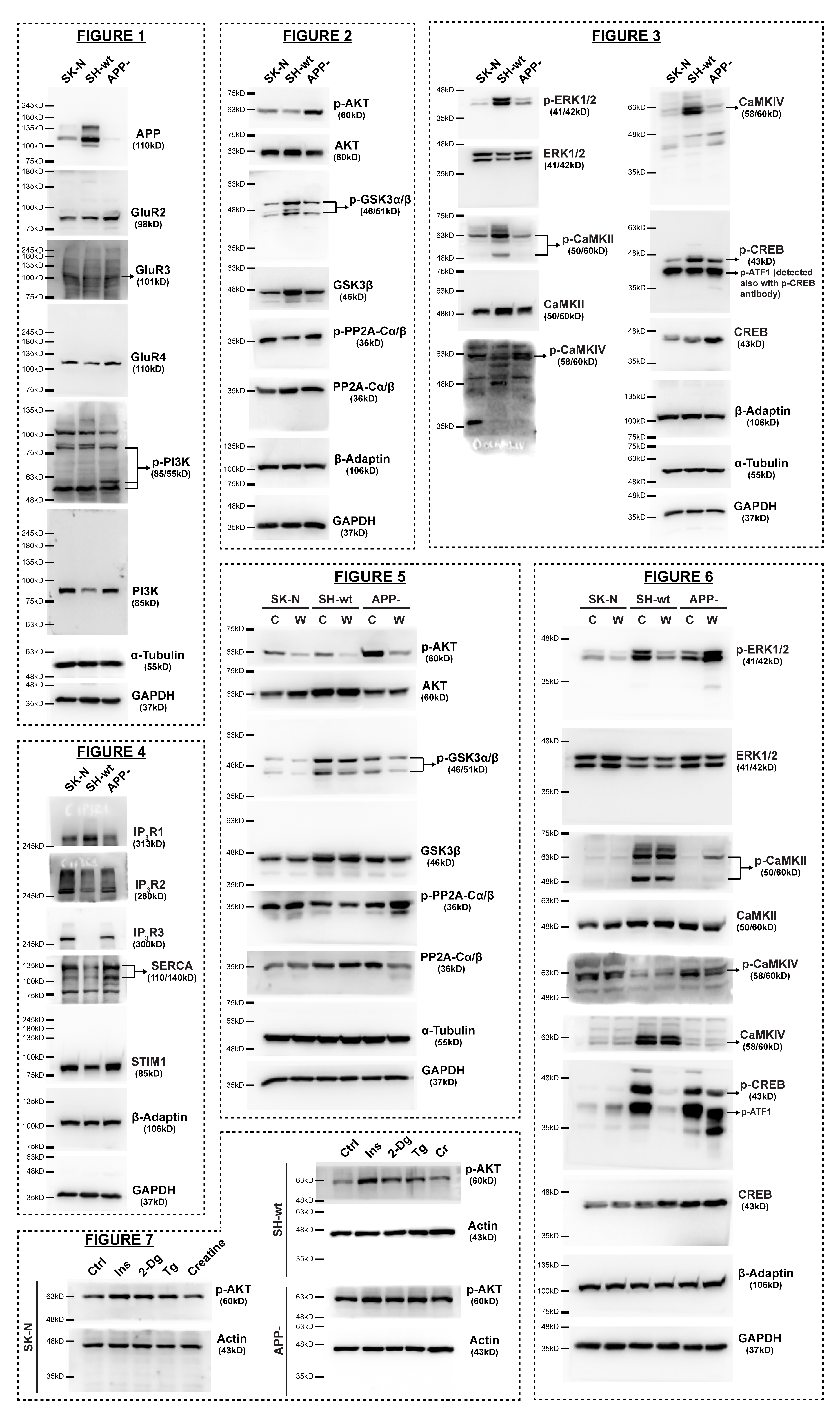

Supplement: Supplementary file 1 [file ijms-24-02302-s001.zip › Figure S1.tif]
